# Myocardial tissue characterisation with late gadolinium enhancement in rheumatoid arthritis, systemic lupus erythematosus and systemic sclerosis

**DOI:** 10.1186/1532-429X-15-S1-O47

**Published:** 2013-01-30

**Authors:** Ntobeko A Ntusi, Jane M Francis, Paul M Matthews, Paul B Wordsworth, Stefan Neubauer, Theodoros Karamitsos

**Affiliations:** 1 OCMR, Cardiovascular Medicine, University of Oxford, Oxford, UK; 2 GSK Clinical Imaging Centre, Imperial College, London, UK; 3 Bortnar Institute and Nuffield Department of Orthopaedics, Rheumatology and Musculoskeletal Sciences, University of Oxford, Oxford, UK

## Background

Rheumatoid arthritis (RA), systemic lupus erythematosus (SLE) and systemic sclerosis (SSc) commonly involve the cardiovascular system, and are associated with high morbidity and mortality. Mechanisms of cardiovascular disease (CVD) involvement in these clinical entities are not fully understood. Furthermore, little is known about myocardial structure and function in these inflammatory arthropathies. Late gadolinium enhancement (LGE) cardiovascular magnetic resonance (CMR) imaging is a tool for noninvasive evaluation of myocardial fibrosis that has the advantage over other imaging techniques of being able to directly visualise both ischaemic and non-ischaemic patterns of injury, and has prognostic significance. The purpose of this study was to assess the frequency and pattern of LGE in RA, SLE and SSc patients without any known CVD using CMR and to determine its relation to disease duration, vascular function (aortic distensibility; pulse wave velocity) and left ventricular (LV) systolic function (LV ejection fraction; mid short axis circumferential systolic strain).

## Methods

59 RA patients (42 female, mean age 53 ± 12), 29 SLE patients (28 female, mean age 42 ± 10), 18 SSc patients (17 female, mean age 55 ± 10), 45 normal controls (38 female, mean age 44 ± 12), and 14 controls with cardiovascular risk factors [CVRFs] (11 female, mean age 52 ± 8) underwent CMR at 1.5 Tesla. All patients with previously known CVD were excluded. Biventricular volumes and function, presence and pattern of LGE, myocardial strain and vascular function were assessed by CMR.

## Results

LGE was found to occur in 46% of RA, 31% of SLE and 50% of SSc patients compared to 9% of normal controls and 21% of controls with CVRFs (p<0.001). The patterns of LGE are presented in Table 1. Presence of LGE correlated with disease duration (Rs= 0.33; p<0.001), myocardial strain (Rs= 0.29; p<0.001), and total aortic pulse wave velocity (Rs= 0.72; p<0.001).

**Table 1:** Frequency and patterns of LGE in RA, SLE and SSc patients and in normal controls and controls with CVRFs.

	Normal controls N=45	Controls with CVRFs N=14	RA N=59	SLE N=29	SSC N=18	P Value
Frequency of LGE	4 (8.9)	3 (21.4)	27 (45.8)	9 (31.0)	9 (50.0)	<0.001

Pattern of LGE Basal inferolateral LGE Lateral wall midwall/subepicardial LGE Septal midwall LGE Myocardial infarction	1 (2.2) 1 (2.2) 2 (4.4) 0 (0)	2 (14.3) 0 (0) 1 (7.1) 0 (0)	16 (27.1) 5 (8.5) 3 (5.1) 3 (5.1)	5 (17.2) 2 (6.9) 0 (0) 2 (6.9)	4 (22.2) 5 (27.8) 0 (0) 0 (0)	<0.001 <0.001 <0.001 <0.001

## Conclusions

CMR demonstrates an increased burden of both ischaemic and non-ischaemic fibrosis in patients with inflammatory systemic diseases with no known CVD. Increased myocardial fibrosis may contribute to the poor cardiovascular outcomes in this group of patients. LGE in RA, SLE and SSc correlates with increasing disease duration, impaired myocardial strain, and increased pulse wave velocity.

## Funding

NN is funded by the Discovery Foundation and Nuffield Trust. SN acknowledges support from the British Heart Foundation Centre of Research Excellence, Oxford. The research was funded through an investigator-led grant from GSK.

**Figure 1:**
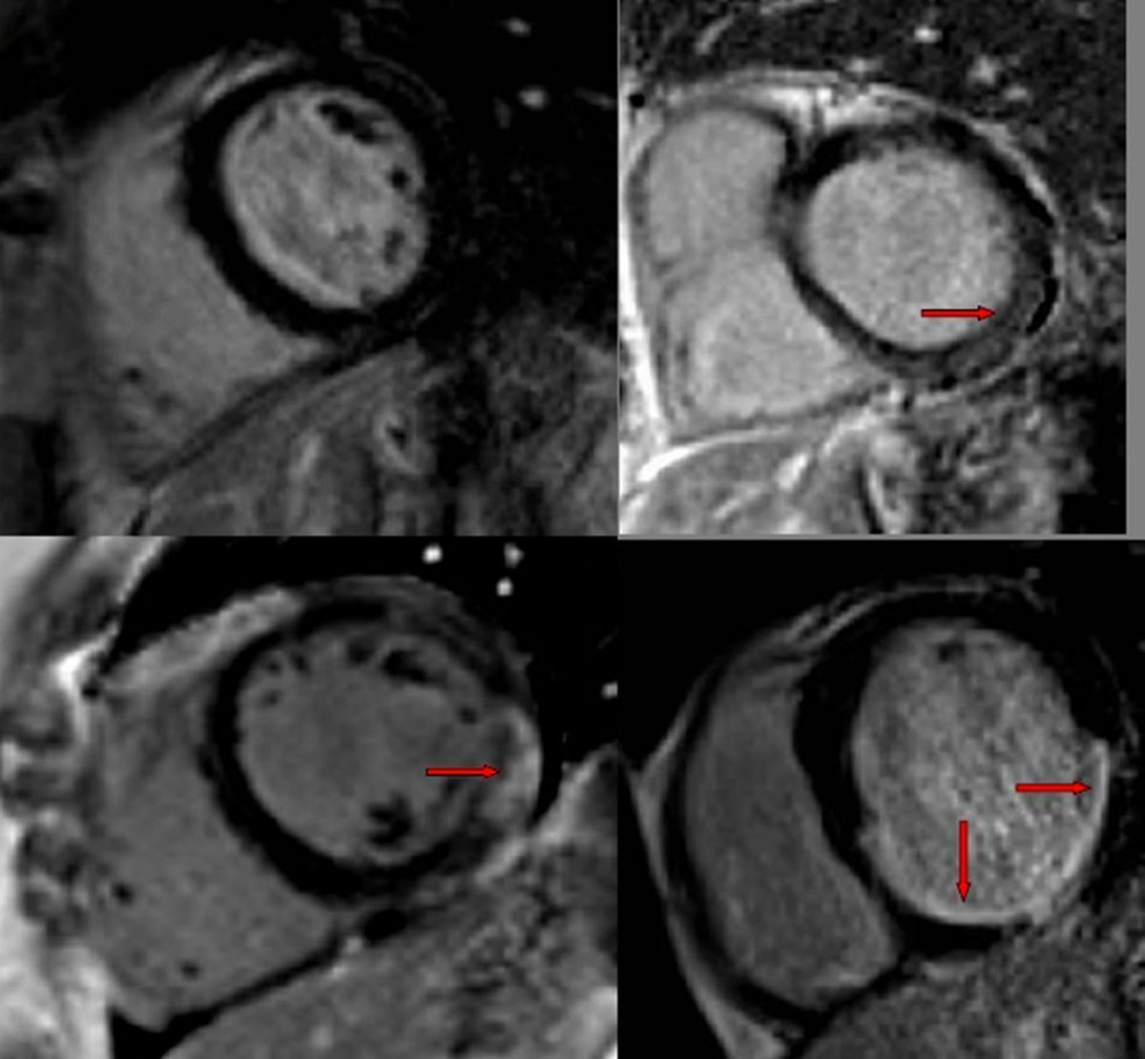
Different patterns of LGE in patients with inflammatory arthropathies (Top left: control with no LGE; top right: SSc patient with basal inferolateral wall LGE; bottom left: SLE patient with lateral midwall/subepicardial LGE; bottom right: RA patient with myocardial infarction (subendocardial LGE).

